# Incidence of Subsequent Cholangiocarcinomas After Another Malignancy: Trends in a Population-Based Study

**DOI:** 10.1097/MD.0000000000000596

**Published:** 2015-02-27

**Authors:** Kai Mao, Wen Jiang, Jieqiong Liu, Jie Wang

**Affiliations:** From the Department of Hepatobiliary Surgery (KM, JW), Sun Yat-sen Memorial Hospital, Sun Yat-sen University, Guangzhou, China; Department of Medicine (KM), Johns Hopkins University, Baltimore, Maryland; Department of Radiation Oncology (WJ), MD Anderson Cancer Center, Houston, Texas; Department of Breast Surgery (JL), Breast Tumor Center, Sun Yat-sen Memorial Hospital, Sun Yat-sen University, Guangzhou, China; and Department of Surgery (JL), Johns Hopkins University, Baltimore, Maryland.

## Abstract

Cholangiocarcinoma (CCA) characterized by late diagnosis and poor outcomes represents the commonest malignancy of biliary tract. Understanding metachronous cancer associations may achieve earlier detection. We aimed to evaluate the risk of subsequent CCAs among common cancer survivors.

The National Cancer Institute's Surveillance, Epidemiology, and End Results database (1973–2010) was reviewed for patients with 1 of the 25 primary cancers. Standardized incidence ratios (SIRs) were calculated as an approximation of relative risk for subsequent CCAs after primary malignancy. Data were stratified by age at primary cancer diagnosis, latency period, and application of radiation.

A total of 1487 patients developed subsequent CCAs. For patients diagnosed with primary cancers between the ages 20 and 39 years, the risk was increased among colon (SIR 14.65), gallbladder (129.29), and uterus (7.29) cancer survivors. At ages of 40 to 59 years, oral cavity and pharynx (1.89), stomach (3.24), colon (1.76), gallbladder (11.78), and lung cancers (1.75) were associated with increased risk. We found persistently elevated SIRs after colon and gallbladder cancer between ages 60 and 79 years. The SIR remained significant among gallbladder cancer survivors diagnosed after 80 years. Gallbladder cancer showed elevated risk at all of the latency periods except first 6 to 11 months. Increased risk of lung cancer (1.66) was detected after 120 months. However, radiation therapy did not contribute to increased risk.

This population-based study suggests that several initial cancers are associated with elevated risk of CCA. The increased risk may be due to shared genetic or environmental etiological factors between these malignancies. Lower threshold for CCA surveillance may be warranted in high-risk patients.

## INTRODUCTION

Cholangiocarcinoma (CCA) is the commonest malignancy of the biliary tract and the second most common hepatic malignancy after hepatocellular carcinoma (HCC).^1^ CCA can be classified anatomically as intrahepatic, perihilar, and distal CCA, and the mean age at diagnosis is 50 years.^2^ The overall incidence of CCA has increased universally over the past 30 years.^3^ In the United States, age-adjusted rates of CCA are reported to be the highest in Hispanic and Asian people (2.8–3.3 per 100,000) and lowest in non-Hispanic white population and black population (both 2.1 per 100,000).^4,5^ Increased incidence has contributed to elevated interest in this cancer; however, the current 5-year survival rate is still very low, ∼10%.^6^ Therefore, early detection of CCA might allow for better outcomes through treating the cancer at an earlier stage. Well-known risk factors for CCA include sclerosing cholangitis, biliary duct cysts, hepatolithiasis, cirrhosis, viral hepatitis C and B, diabetes, as well as hepatobiliary flukes.^7–13^ Recent efforts in the epidemiology of CCA have focused on the role of several genes in the genetic transformation of this cancer. These CCA-associated genes could be classified into those encoding proteins regulating DNA repair (methylenetetrahydrofolate reductase, thymidylate synthetase, glutathione S-transferase ω-1, and x-ray repair cross-complementing protein 1), cellular protection against toxins (adenosine triphosphate-binding cassette subfamily C member 2, cytochrome P450 1A2, and *N*-acetyltransferase 2 [NAT2]), and immunological surveillance (killer cell lectin-like receptor subfamily K, member 1; major histocompatibility complex class I chain-related gene A; prostaglandin-endoperoxide synthase 2).^6^ However, much remains to be learned regarding the etiology of CCA. For example, the results of studies on the role of smoking and alcohol exposure were inconsistent.^6,10^ Identifying people at risk for CCA may be an effective way to achieve earlier detection and higher survival rates.

So far, no population-based study has evaluated the risk of a second primary CCA in patients with common cancers. Thus, the purpose of the current study was to systematically investigate whether common primary malignancies are associated with an increased risk of subsequent CCA, with a particular emphasis on determining how the risk varies with latency periods or diagnostic age of the initial primary cancer.

## METHODS

The National Cancer Institute's Surveillance, Epidemiology, and End Results (SEER) program was used as the source of patient information for this study. The SEER program collects cancer incidence and survival data from population-based cancer registries across the United States. To allow for enough follow-up time, our study included patients from the original SEER 9 registry database.^14^ Data were available for cases diagnosed from 1973 through 2010, and collectively cover ∼10% of the US population.

In this study, we focused mainly on the 25 commonly diagnosed cancers including oral cavity and pharynx, esophagus, stomach, colon, rectum, liver, gallbladder, pancreas, retroperitoneum, larynx, lung, bones and joints, soft tissue, skin melanoma, female breast, cervix uteri, uterus, ovary, prostate, urinary bladder, kidney, renal pelvis, nervous system, thyroid, lymphoma, and leukemia. We included all cancers mentioned above in patients with age >20 years old that were pathologically confirmed. And we excluded death certificate-only and autopsy-only cases. Cases diagnosed within 6 months of the initial primary tumor were also excluded because these were likely to be preexisting or synchronous cancers. According to the inclusion criteria above, a cohort with any of these 25 common cancers was followed up, and subsequent CCA was observed. Diagnosis of subsequent CCA, death, or censoring by December 31, 2010 was defined as end event.

To compare the relative risk with the general population in the 9 SEER areas, we used SEER^∗^(software provided by Surveillance, Epidemiology, and End Results, Program of the National Cancer Institute of the United States) Stat Multiple Primary-Standardized Incidence Ratios (SIRs) program (version 8.1.5) to calculate SIRs by dividing the observed numbers of subsequent CCA by the expected numbers of subsequent CCA for various primary malignancies (observed/expected), along with their exact 95% confidence interval (CI). Data were stratified by latency periods (6–11, 12–59, 60–119, and 120+ months), age at diagnosis of the first primary malignancy (20–39, 40–59, 60–79, and 80+ years), and application of radiation therapy. All *P* values were 2-sided and considered statistically significant at the *P* < 0.05 level. The Sun Yat-sen Memorial Hospital reviewed this study and declared it exempt because of a lack of protected health information contained in the databases used. Moreover, no consent was needed in this study.

## RESULTS

The present study included 2,374,060 patients diagnosed with 1 of the 25 most common primary malignancies that met our inclusion criteria. Among these individuals, 1487 (0.06%) were identified with a subsequent CCA at least 6 months after the initial malignances.

**FIGURE 1:**
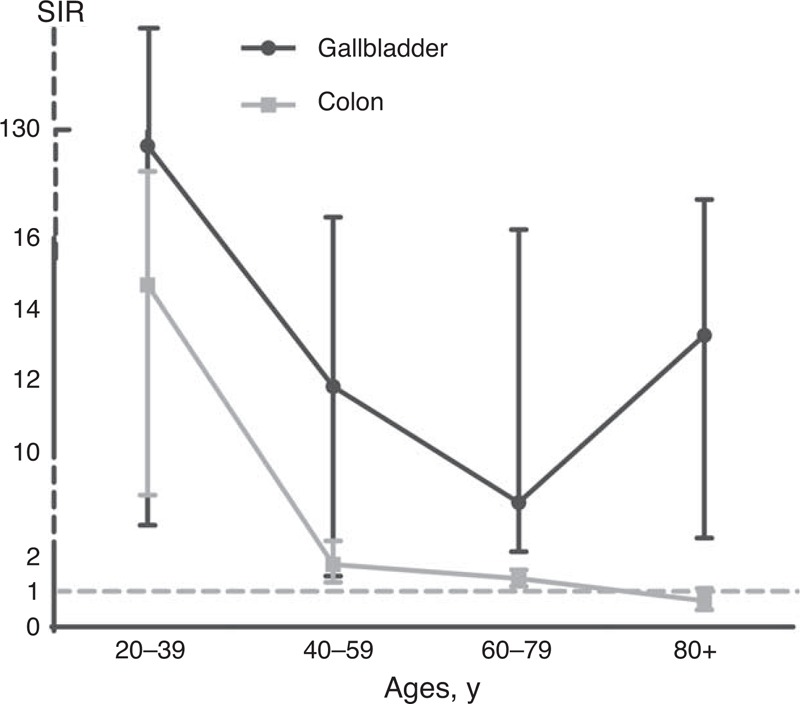
Risk of CCA among survivors of gallbladder and colon cancers diagnosed at different age groups. The error bars indicate the 95% CIs. CCA = cholangiocarcinoma, CI = confidence interval, SIR = standardized incidence ratio.

Analysis stratified by ages at diagnosis revealed that cancers of oral cavity and pharynx, stomach, colon, gallbladder, lung, and uterus had significantly elevated SIRs for subsequent CCA (Table 1). Between the ages of 20 and 39 years, cancers of colon (14.65, 95% CI 6.32–28.86), gallbladder (129.29, 95% CI 3.27–720.34), and uterus (7.29, 95% CI 1.99–18.66) had significantly increased SIRs. Moreover, oral cavity and pharynx (1.89, 95% CI 1.14–2.95), stomach (3.24, 95% CI 1.30–6.67), colon (1.76, 95% CI 1.25–2.42), gallbladder (11.78, 95% CI 1.43–42.57), and lung cancers (1.75, 95% CI 1.02–2.80) were associated with significantly increased risk of the second CCA at the ages of 40 to 59 years. Among individuals diagnosed with an initial malignancy between the ages of 60 and 79 years, we found persistently elevated SIRs for subsequent CCA after colon cancer (1.36, 95% CI 1.14–1.60) and gallbladder cancer (8.50, 95% CI 3.42–17.52). Among patients with a primary cancer diagnosed after age 80 years, the SIR remained elevated for gallbladder cancer (13.23, 95% CI 3.6–33.87). We observed that patients with gallbladder cancer had significantly elevated risk of subsequent CCA at all of the 4 age groups, and colon cancer was associated with increased risk at the ages <80 years (except 80+ year age group). Both of gallbladder cancer and colon cancer had a prominent decrease in SIRs for second primary CCA, which revealed that age at diagnosis of the primary cancer seemed to play a role in the associations between CCA and these malignancies (Figure 1).

**TABLE 1:**
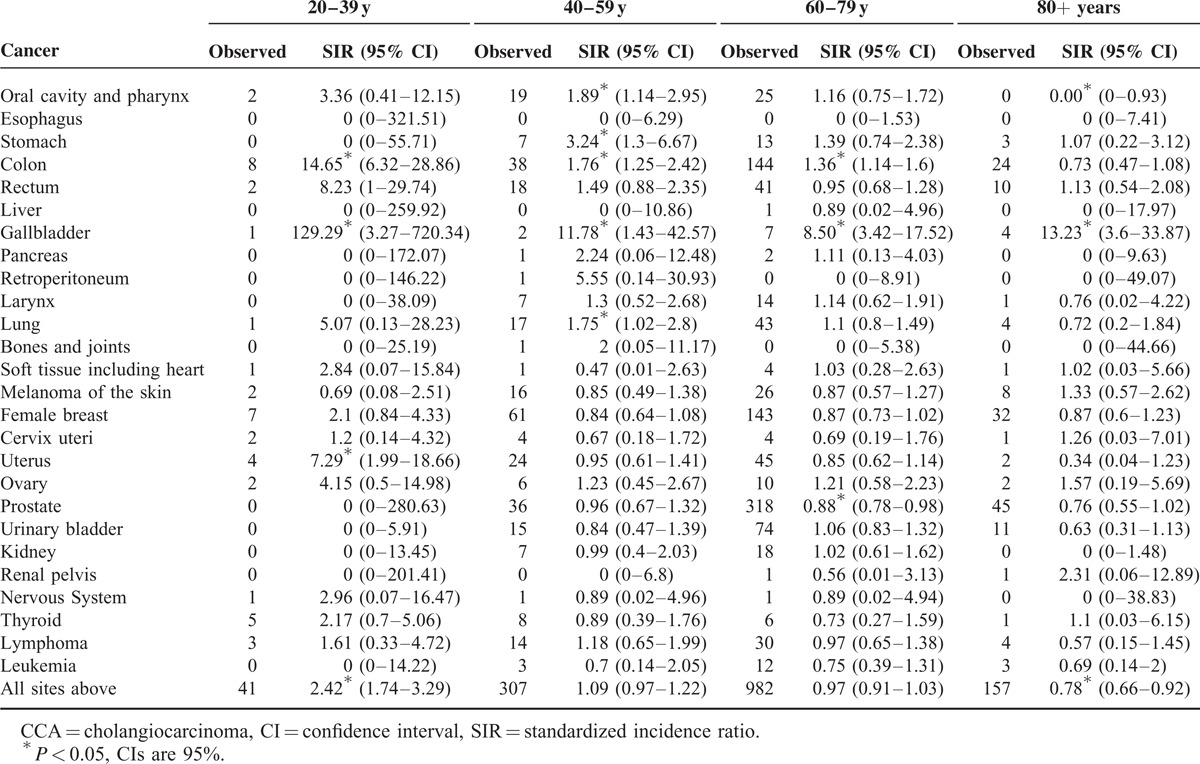
Risk of Subsequent CCAs After 25 Common Primary Cancers, Stratified by Ages

In term of latency period, the risk of subsequent CCA was increased after several primary malignancies (Table 2). Among patients diagnosed between the latency of 12 and 59 months, stomach (2.32, 95% CI 1.23–3.96) and gallbladder (11.59, 95% CI 4.25–25.23) were associated with a significantly increased risk of subsequent CCA. At the latency of 60 to 119 months, the risk of subsequent CCA was significantly elevated after colon cancer (1.54, 95% CI 1.20–1.96) and gallbladder cancer (13.00, 95% CI 3.54–33.29). At the latency of 120+ months, the increased risk of subsequent CCA for gallbladder cancer (9.17, 95% CI 1.89–26.79) and lung cancer (1.66, 95% CI 1.00–2.60) were detected. No initial malignancy associated with significantly elevated SIR of subsequent CCA was observed at the latency of 6 to 11 months.

**TABLE 2:**
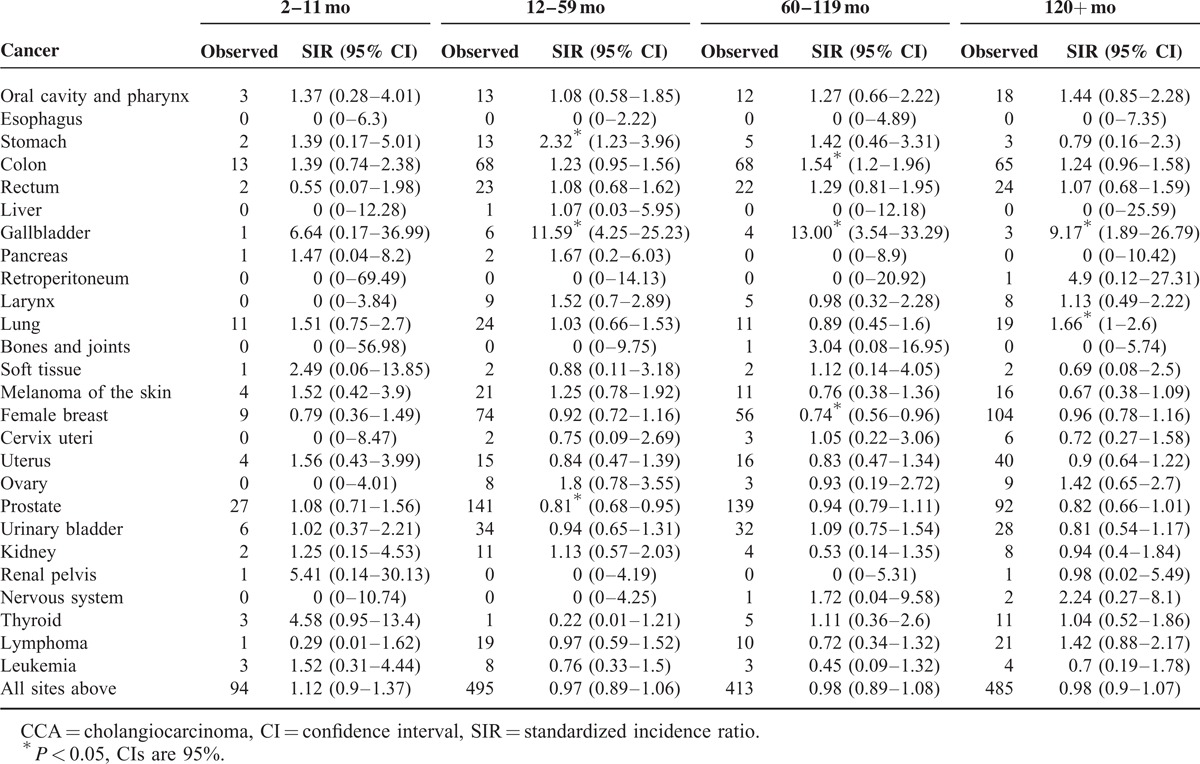
Risk of Subsequent CCAs After 25 Common Primary Cancers, Stratified by Latency Periods

We further evaluated the effects of radiation therapy during treatment of the first primary malignancy (lung and gallbladder cancers). Subset analysis showed that the risk of subsequent CCA was increased in the nonradiation-treated group, whereas there was no significant association between CCA and lung or gallbladder cancer among the patients who underwent radiation treatment (Table 3).

**TABLE 3:**
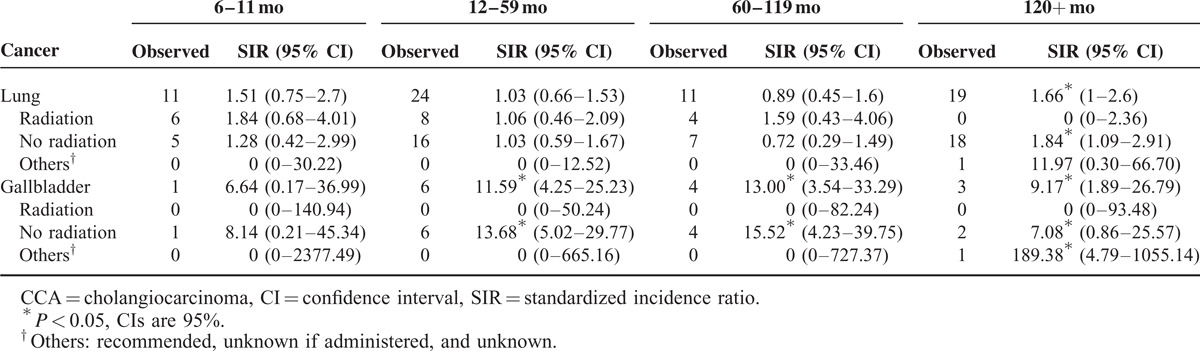
Risk of Subsequent CCAs After Lung and Gallbladder Cancers, Stratified by Application of Radiation Therapy

## DISCUSSION

Common epidemiologic risk factors such as genetic susceptibility, environmental exposure, and lifestyle are shared between different malignancies; the hypothesis that a primary malignancy is associated with increased risk of a second primary cancer has been confirmed by lots of studies. Amin et al^15^ found that hepatobiliary carcinoma was associated with an elevated overall risk of a subsequent pancreatic cancer. However, to the best of our knowledge, there is no population-based study focusing on the incidence of subsequent CCA after another malignancy. The current study filled the gap in this field, and our analysis is novel because we use the population-based dataset US SEER to evaluate the short- and long-latency courses of the risks rather than overall risk in the associated cancers, and we detected how the risk varies with age at onset of the primary cancer.

We identified increased risk of developing a subsequent CCA among primary gallbladder cancer survivors almost in all age groups and latency periods. Gallbladder cancer is the initial primary cancer with the largest SIR in the current study. This could be explained by shared well-known risk factors due to the special anatomy such as sclerosing cholangitis between these 2 malignancies^16,17^ and the same cancer-associated genes such as kirsten rat sarcoma viral oncogene homolog, tumor protein p53, NAT2, and human epidermal growth factor receptor 2.^6,18–21^ We also observed significant associations between colon, stomach, uterus (endometrial) cancers, and CCA, especially in younger age groups. Many cancers, particularly in individuals at younger age, can be a result of several genetic syndromes, such as hereditary breast–ovarian cancer syndrome and hereditary nonpolyposis colon cancer (HNPCC) syndrome.^22,23^ Thus, possible explanation of this age-specific pattern is that these 4 carcinomas all belong to HNPCC syndrome, also known as Lynch syndrome.^23^ As we know, HNPCC syndrome also includes pancreatic cancer, and due to the specific anatomy choledochopancreatic duct, there might be an increased risk of CCA after primary pancreatic cancer. Moreover, due to shared etiology (cirrhosis and viral hepatitis C and B) between HCC and CCA, hepatocellular cancer may be associated with CCA as well. However, we failed to find significant elevated risks of CCA among survivors of pancreatic or liver cancer. We consider that this result might be due to the significant poor prognosis of pancreatic cancer and HCC, with a very low 3-year overall survival of 4% to 6% and 24%, respectively.^24,25^ Chakraborty et al^26^ reported an SIR of 1.42 for the development of hepatobiliary carcinoma after a primary renal cancer. Our data showed no statistical risk of developing CCA after renal cancer. The result suggested that the elevated SIR of subsequent hepatobiliary cancer among renal cancer survivors may be contributed by increased risk of subsequent gallbladder or hepatic carcinomas. Further study is required to shed light on this situation.

Although data of the effect of smoking exposure on the carcinogenesis of CCA were not consistent,^6,10^ we detected increased incidences of CCA after other malignancies (stomach, lung, oral cavity, and pharynx cancers) known to be related with tobacco consumption between the ages of 40 and 59 years. The risk trend of primary stomach cancer also occurred in the 12 to 59 months latency period, and a significant association between lung cancer and CCA was found in the >10-year latency period. Unfortunately, smoking history is not included in the SEER database, and it was impossible for us to evaluate whether the tobacco usage could increase the risk of a second primary CCA.

As mentioned above, an elevated risk of CCA was shown in patients with primary lung or gallbladder cancer beyond 10 years after diagnosis. This might be partly explained by smoking exposure of patients or genetic susceptibility and shared risk factors, but certain observations warrant cautious consideration. Radiation therapy of the primary cancer may induce second primary malignancy in the long latency period (>10 years after radiation),^27^ so we further performed subset analysis by evaluating the risks of developing CCA in patients with primary lung or gallbladder cancer with or without radiation therapy. Although the risk was increased in the nonradiation-treated group, there was no significant association between CCA and lung or gallbladder cancer in the patients who underwent radiation treatment. The results illustrated that radiation treatment failed to play a role in the increased incidence of CCA followed by primary lung or gallbladder cancer. Given that radiation is typically delivered in the adjuvant setting or as a component of definitive treatments for more advanced stage diseases, the lack of observation of increased secondary CCA risk among lung or gallbladder cancer survivors with previous radiotherapy may be explained by the heavier tumor burden of these patients. Thus, patients who underwent radiotherapy may in fact experience worse survival that precludes the development of subsequent CCA.

The primary strength of this study is the large number of patients derived from population-based registries with the 37-year period of data collection. Another strength is the in-depth assessment of the effects of latency period, diagnostic age, and application of radiation therapy on risks. Weaknesses of the SEER database include lack of tobacco, alcohol intake, or BMI information; lack of family history and comorbidities of patients; as well as retrospective nature of the data collection. Additionally, possible bias toward identifying CCA in patients already diagnosed with other biliary duct carcinomas might exist.

Nonetheless, our results demonstrated that the risk of subsequent CCA was increased after several primary malignancies that share similar genetic, epidemiological, and exposure risk factors. Such clear recognition of the association between CCA and other cancers may improve the national CCA surveillance protocol (lower threshold for patients with high risks) and finally help to prolong the overall survival via earlier detection. However, additional studies with more detailed information are needed to further investigate the underlying biological and environmental mechanisms.
